# Correction: Zhang et al. Clinical Management of Low Anterior Resection Syndrome: Review of the Current Diagnosis and Treatment. *Cancers* 2023, *15*, 5011

**DOI:** 10.3390/cancers16020459

**Published:** 2024-01-22

**Authors:** Ruijia Zhang, Wenqin Luo, Yulin Qiu, Fan Chen, Dakui Luo, Yufei Yang, Weijing He, Qingguo Li, Xinxiang Li

**Affiliations:** 1 Department of Colorectal Surgery, Fudan University Shanghai Cancer Center, Shanghai 200032, China; 17301050248@fudan.edu.cn (R.Z.); 21111230037@m.fudan.edu.cn (W.L.); 19301050224@fudan.edu.cn (Y.Q.); 19301050220@fudan.edu.cn (F.C.); dkluo17@fudan.edu.cn (D.L.); 20111230052@fudan.edu.cn (Y.Y.); wjhe15@fudan.edu.cn (W.H.); 2 Department of Oncology, Shanghai Medical College, Fudan University, Shanghai 200032, China

## Additional Affiliation(s)

In the published publication [1], there was an error regarding the affiliation(s) of all authors. In addition to the affiliation “^2^ Department of Oncology, Shanghai Medical College, Fudan University, Shanghai 200032, China”, the updated affiliation(s) should include: “^1^ Department of Colorectal Surgery, Fudan University Shanghai Cancer Center, Shanghai 200032, China”. The authors state that the scientific conclusions are unaffected. This correction was approved by the Academic Editor. The original publication has also been updated.
